# Interpretable machine learning via symbolic classification of radiomic texture and morphological features for pediatric pneumonia detection from chest X-rays

**DOI:** 10.1371/journal.pone.0351081

**Published:** 2026-06-22

**Authors:** Filippos Sofos, Zoi D. Pana, Dimitris Drikakis

**Affiliations:** 1 Institute for Advanced Modelling and Simulation, University of Nicosia, Nicosia, Cyprus; 2 Condensed Matter Physics Laboratory, Department of Physics, University of Thessaly, Lamia, Greece; 3 Department of Basic and Clinical Studies, University of Nicosia, Nicosia, Cyprus; University of Pisa, ITALY

## Abstract

Explainable artificial intelligence in medical imaging is currently dominated by post-hoc tools that rationalise the decisions of otherwise opaque deep networks, without providing, most of the time, a robust and transparent decision rule. This paper presents an interpretable mathematical model for pneumonia detection in pediatric chest radiographs. We propose a symbolic classification framework that evolves a non-linear closed-form diagnostic formula directly from a compact set of clinically grounded radiomic markers, including entropy, solidity, and fractal dimension. To our knowledge, this is the first single-formula symbolic classifier reported for pediatric pneumonia detection on the specific dataset. The symbolic classifier achieved 87% accuracy and AUC = 0.93 under 10-fold cross-validation. When the selected closed-form equation was applied to the filtered independent hold-out test set, it achieved 79.1% accuracy and AUC = 0.89. The equation has been further validated and re-calibrated on an independently acquired external dataset. With a parameter count several orders of magnitude smaller than that of competing deep learning models, and an auditable closed-form expression, the proposed model provides a lightweight, transparent baseline suited to resource-constrained inference and regulatory audit. The proposed framework can be applied in complementary ways to existing deep learning pipelines, as an intrinsically interpretable alternative that broadens the methodological repertoire for clinically transparent diagnosis.

## Introduction

Contemporary diagnostic frameworks are increasingly integrating machine learning (ML) and deep learning (DL) to map complex imaging and/or textual clinical parameters to specific pathological markers [1]. Chest X-ray (CXR) radiographs have been widely utilised as an accessible clinical tool for diagnosing pneumonia. However, interpreting these images is challenging even for experienced radiologists, as the visual evidence of pneumonia is often ambiguous and can easily be mistaken for other pulmonary pathologies [2]. As a consequence, there is an urgent need for robust computer-aided diagnosis systems to assist clinicians in achieving more accurate and consistent pneumonia detection. To this end, advanced data-driven models have been proposed that move beyond traditional manual radiological assessment.

Clinical datasets and advanced ML/DL techniques have been combined in several related works. For instance, in [3], the ChestNet architecture was proposed as a problem-based architecture for pediatric radiographs, achieving an accuracy of 94.67%. The authors note that the system is limited to detecting focal consolidation and cannot interpret clinical significance without integrating additional clinical data. In [4], a weighted ensemble of three CNN models, GoogLeNet, ResNet-18, and DenseNet-121, has been utilised to achieve pneumonia detection accuracy rates of 98.81% and 86.85% on the CXR and the Radiological Society of North America (RSNA) datasets [5], respectively. However, the framework is limited by high computational costs and is prone to misclassifying images with poor contrast or subtle, early-stage pneumonia infiltrates. High accuracy and complexity, achieved through transfer learning and CNN-based architectures, have also been reported in [6] and [7].

On the other hand, more complex DL architectures are required when disease identification becomes multi-decisive. For the NIH ChestX-ray14 dataset, the infections refer to cases beyond binary “Normal” and “Pneumonia”, such as “Atelectasis”, “Cardiomegaly”, “Effusion”, “Infiltration”, “Mass”, “Nodule”, “Pneumonia” and “Pneumathorax” [8]. A small portion of this dataset was also used to construct a traditional five-algorithm ML classifier and apply it to segmented lung regions [9]. It is noted that this approach outperforms processing the full CXR images. Techniques that enhance CXR images before feeding them into the DL network have also been proposed [10–14]. A broader list of research efforts that apply DL methods to detect pneumonia in CXR images can be found in recent reviews [15–19].

Beyond achieving high predictive accuracy, the interpretability of these models is essential to address the ethical, legal, and regulatory requirements inherent in modern medicine [20]. It is a fact that purely black-box models often struggle with the risk of learning noise or artefacts inherent in clinical datasets, which can limit their reliability in a real-world medical environment [21]. Regulatory bodies increasingly mandate transparency and accountability, requiring that Artificial Intelligence (AI) tools provide a clear pathway for their clinical predictions to streamline approval processes. By providing a clear view into the decision-making process, stakeholders can better identify potential biases, validate algorithmic recommendations, and mitigate errors, especially when the system produces unexpected or conflicting results [22].

To address these limitations, it is critical to construct predictive models that remain clinically transparent and explainable [23–26]. By synthesising ensemble learning with explainable AI (XAI), high predictive accuracy and transparent logic are combined to build clinical trust and support collaborative decision-making [27]. Another popular computational suite employs genetic programming (GP) methods [28], such as Symbolic Regression (SR), to discover hidden relationships between dataset features and express them as explicit mathematical formulas [29]. Recent advancements have extended the SR framework to Symbolic Classification (SC), enabling the derivation of high-precision diagnostic indices that map these complex features directly to discrete clinical outcomes [30].

In this paper, the proposed pipeline begins by pre-processing raw clinical pediatric chest radiographs from both healthy subjects and patients with pneumonia to extract physically meaningful texture descriptors. We apply targeted image-processing techniques, including normalisation, denoising, and morphological segmentation, to isolate specific regions of interest in the radiographs. From these regions, we extract a set of high-dimensional texture-based features, including *Entropy*, *Solidity*, *Homogeneity*, *HazeRatio*, and *Fractal Dimension*, as well as key statistical descriptors, such as *Skewness*, *Kurtosis*, and *Contrast*. This deterministic approach ensures that the input data reflects the underlying pathophysiology of pulmonary infection, providing a structured representation of the lung parenchyma that is often lost in fully automated black-box pipelines. This methodology focuses on a novel feature-centric framework that shifts the diagnostic focus from latent pixel patterns to measurable, clinically grounded morphological indicators.

On this basis, this work can be seen as an intrinsic interpretability framework [31], differentiating from popular post-hoc explainability methods. For instance, the Pixel-Level Interpretability Model (PLI) and Gradient-Weighted Class Activation Maps (Grad-CAMs) [32,33] highlight image regions that most affect the classification decision. Moreover, Shapley Additive Explanations (SHAP) assigns additive importance values to pixels to quantify their marginal contribution to detecting lung opacities [34], Local Interpretable Model-agnostic Explanations (LIME) isolates interpretable super-pixels through local perturbations to justify classifications such as cardiomegaly [35], and attention-based saliency, leverages the internal weighting of the model, especially in vision transformers, to visualize the global feature dependencies that currently dominate medical imaging XAI [36]. All these post-hoc methods produce approximations of an otherwise opaque model and have been shown to fail elementary sanity checks [37] and to be susceptible to adversarial manipulation [38].

On the other hand, with a closed-form SC, the model and the explanation come as a single, deterministic mathematical expression that can be verified by direct inspection, re-implemented without any ML library, and audited for regulatory purposes. The SC algorithm derives explicit mathematical relationships that maintain diagnostic performance while ensuring the full transparency required to map clinical findings directly to interpretable image descriptors, replacing opaque pixel-level heuristics. By evolving mathematical expressions directly from the extracted texture and statistical features, a non-linear diagnostic index that remains human-readable is derived. To our knowledge, no prior study has reported a single closed-form SC, evolved directly from radiomic descriptors, for pediatric pneumonia detection on this benchmark, replacing opaque neural weights with a single, deterministic diagnostic formula. Therefore, a mathematical approach to pneumonia is proposed, with a final model that is both accurate and scientifically sound in clinical settings, where interpretability is a prerequisite for adoption.

## Materials and methods

### Dataset preparation and feature extraction

The dataset used in this paper consists of a publicly available collection of pediatric chest X-ray (Kermany Pediatric CXR) images from the Guangzhou Women and Children’s Medical Centre [39]. It comprises high-resolution radiographs categorised into two primary groups, “Normal” and “Pneumonia”, which are further classified as “Bacteria” and “Virus” infections. They provide a realistic clinical environment for testing the accuracy and reliability of the proposed SC model. Images from the Kermany Pediatric CXR dataset are divided by the authors into 5,232 images for training (3,883 pneumonia and 1,349 normal), and the held-out test partition contains 624 images (390 pneumonia and 234 normal).

Inclusion was limited to images acquired during routine clinical care. The original release applied a two-physician quality screen that removed low-quality and unreadable scans prior to publication, and labels were adjudicated by a third expert reviewer for the test partition. Acquisition-protocol details (imaging device make and model, exposure parameters) beyond the use of standard anterior-posterior projection are not reported in the public release, and we did not have access to the underlying DICOM metadata. We acknowledge that the dataset is single-institution and single-demographic (East-Asian pediatric population), that NLP- and expert-derived label noise have been described in the literature for this benchmark [40], and that these factors collectively constitute the principal sources of dataset bias for the present study.

After reading the images from the available Kermany Pediatric CXR dataset, numerical descriptors were computed for each patient’s scan in MATLAB R2025B. These parameters, ranging from simple intensity statistics to complex texture measures, are detailed in Table 1. They act as the parameter space used for the SC pipeline developed later in this study. Images that exhibit an extreme aspect ratio (outside the 0.7–1.5 range, indicative of stretched or non-standard framing) or have poor radiographic quality (i.e., severely under-exposed or near-uniform images with negligible contrast), are excluded from the pipeline that follows. In such a way, our training dataset now consists of $N_{train}=3373$ entries and the test set of $N_{test}=392$ entries.

**Table 1:** Mathematical definitions and clinical significance of extracted radiographic features.

Feature	Symbol	Definition	Clinical Significance
EntropyMean	$H_{\mu }$	$\frac{1}{N}\sum_{i=1}^{N}H_{i}$	Global textural disorder and randomness
EntropyMax	$H_{max}$	$\max(H_{i})$	Peak localized complexity in consolidations
Entropy90	*H*_90_	$P_{90}(H_{i})$	High-intensity textural heterogeneity
HazeRatio	*Hz*	$\frac{1}{n}\sum_{x\in \Omega }\min_{c}(I^{c}(x))$	Ground-glass opacities
Solidity	*S*	$\frac{1}{n}\sum [I(x)>0.85]$	Intensity-based density proxy
Contrast^a^	*Con*	$\sum_{i,j}|i-j{|}^{2}p(i,j)$	Vessel vs. infection sharpness
Homogeneity^a^	*Hom*	$\sum_{i,j}\frac{p(i,j)}{1+|i-j|}$	Parenchymal patchiness
Skewness	$\gamma_{1}$	$E[(X-\mu {)}^{3}]/\sigma^{3}$	Histogram asymmetry
Kurtosis	$\gamma_{2}$	$E[(X-\mu {)}^{4}]/\sigma^{4}$	Intensity outlier detection
Fractal Dimension	$D_{f}$	$\lim_{\epsilon \to 0}\frac{\log N(\epsilon )}{\log(1/\epsilon )}$	Structural heterogeneity and complexity

**Note:** *p*(*i*,*j*) is the joint probability of intensity levels *i* and *j*; *P*_90_ represents the $90^{th}$ percentile operator; *N* is the number of intensity levels; $I^{c}(x)$ is the color channel intensity; μ and σ are the mean and standard deviation of intensities; *E* is the expected value operator; N(ϵ) is the number of boxes of size ϵ required to cover the image texture.

^a^Calculated using the grey-Level Co-occurrence Matrix (GLCM) at a displacement of one pixel.

The extraction process begins with the first-order statistical moments of the density histogram. *Skewness* ($\gamma_{1}$) measures the degree of asymmetry in the pixel intensity distribution. A positive skew (i.e., consolidation) indicates a right-tailed distribution, often reflecting localised high-intensity opacities. *Kurtosis* ($\gamma_{2}$) describes the flatness of the distribution, used to identify the presence of extreme outlier values relative to the mean. *Kurtosis* rises in the presence of a small number of extremely dense foci, which is again consistent with focal pneumonic findings [41]. In the context of pulmonary imaging, $\gamma_{1}$ and $\gamma_{2}$ are established representative features for differentiating between various lung disease states, as they capture possible changes in tissue density and heterogeneity that may not be immediately apparent through visual inspection [42].

The texture of the affected (or unaffected) lungs is further analysed using information measures. Pneumonic consolidation and ground-glass opacification disrupt the otherwise quasi-periodic alveolar texture of the healthy lung field and replace it with a heterogeneous grey-level distribution. Shannon entropy of the intensity histogram is the canonical scalar summary of this heterogeneity, and increases monotonically with textural disorder. More specifically, *Entropy* (Mean, Max, and 90^th^ percentile) quantifies the inherent randomness within the image texture, where higher entropy typically correlates with the heterogeneous patterns found in infected lung cases [43]. The *HazeRatio* parameter is employed to quantify local light scattering, which is particularly effective in identifying ground-glass opacities that appear as foggy regions in radiographs [44]. In contrast to other radiomic features, *Fractal Dimension* ($D_{f}$) captures the structural complexity, calculated using the box-counting method with a scaling range of 2–64 pixels to measure how a texture pattern fills the two-dimensional space [45]. The final value represents the slope of the log-log regression, thereby capturing multi-scale textural heterogeneity in the lung parenchyma.

Pulmonary opacification is quantified by a density proxy we denote *Solidity*, calculated as the proportion of pixels in the lung field whose normalised intensity exceeds 0.85. High values therefore correspond to large lung-field areas whose attenuation approaches that of the mediastinum, the radiographic signature of consolidation typical of bacterial pneumonia [46].

Finally, 2^nd^-order statistics are derived from the grey-Level Co-occurrence Matrix (GLCM), a statistical method for texture analysis that quantifies the spatial distribution of pixel intensities [47,48]. It functions by calculating the frequency with which pairs of pixels with specific grey-level values occur at a defined distance and orientation across the image. By capturing these recurring spatial relationships, the GLCM transforms raw pixel data into second-order statistical descriptors, such as *Contrast* and *Homogeneity*, which are essential for identifying complex pathological textures in clinical radiographs. *Contrast* rises and *Homogeneity* falls in regions of consolidation, where the boundary between airless tissue and adjacent residual aerated lung produces sharp local intensity steps.

This radiomic feature set has been chosen over learned representations (e.g., convolutional feature maps) to emphasise interpretability, which is the goal of this study. For a clinician who makes a prediction, it is important to reason about both the model formula and its inputs in radiological terms. Learned representations, by construction, do not satisfy the latter requirement, and their use would shift the interpretability burden from the model to a separate post-hoc explanation method (e.g., saliency maps), which is much different from SC. In such a way, each feature in Table 1 is a measurable image property and a clinically named radiological sign, in parallel.

**Fig 1:**
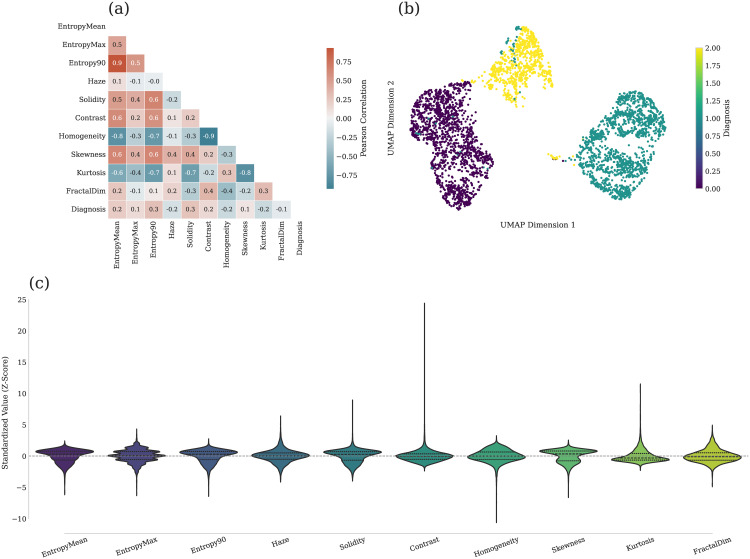
Statistical and topological characterisation of the radiomic feature space. **(a)** Pearson correlation matrix, **(b)** UMAP manifold projection (2D projection of 10 feature space) colour-coded by diagnostic label (Normal, Bacterial, Virus). **(c)** Standardised *Z*-score distributions (violin plots).

The triangular heatmap in Fig 1(a) is the Pearson Correlation Matrix for the image-derived features. Strong positive relationships are observed between *EntropyMean* and *Entropy90* (r≈0.9), suggesting significant redundancy in these texture-based metrics. On the other hand, a significant inverse correlation value exists between *Homogeneity* and *Contrast* (r=−0.9), suggesting that as local image intensity variation (contrast) increases, the uniformity of the pixel distribution (homogeneity) decreases. As far as the output target (*Diagnosis*) is concerned, only relatively weak linear correlations with input features are observed (ranging from −0.2 to 0.3), justifying the use of non-linear approaches to uncover the relations between them.

The Uniform Manifold Approximation and Projection (UMAP) [49] is employed to visualise the high-dimensional structure of the data. This technique performs dimensionality reduction on high-dimensional data (e.g., biological data [50]) by projecting a multi-featured input into a 2D space while preserving the local and global connectivity of the data points. Fig 1(b) reveals three distinct, well-defined clusters. This clear spatial segregation indicates that the extracted textural and density-based features (i.e., the interactions among entropy, solidity, and structural complexity) can characterise the unique phenotypic signatures of “Normal”, “Bacterial”, and “Viral” pneumonia.

The bottom panel (Fig 1(c)) presents feature-specific violin plots of the standardised values (*Z*-Scores), enabling a direct comparison of distributional shapes and outlier profiles. Features such as *EntropyMean* and *Skewness* exhibit tight, symmetric distributions centred around the mean, suggesting they are relatively stable across the patient cohort. *Contrast* and *Kurtosis* exhibit long, thin tails extending above the mean, indicating outlier cases in which specific images exhibit high localised intensity changes. These extreme values may serve as key indicators for identifying severe pathology. Moreover, the observed multimodality in features such as *Solidity* and *EntropyMax* suggests the presence of distinct diagnostic sub-populations, thereby motivating the search for a non-linear classifier that can integrate these heterogeneous distributional shapes into a single diagnostic score.

### Image-to-feature pipeline

A feature-extraction architecture has been developed to transform raw Kermany Pediatric CXR data into a high-dimensional radiomic space that captures subtle textural signatures of pulmonary pathology. This process comprises three critical phases: clinical data validation, localised texture quantification, and structural complexity analysis, as described in Algorithm 1. Initially, by applying geometric filtering and radiometric validation ($\mu_{I}\geq 10$), the pre-processing code removes low-quality artefacts that could introduce bias into the classification code. All images are resized to common dimensions (1024×1024 pixels). Furthermore, Contrast Limited Adaptive Histogram Equalisation (CLAHE) is applied to enhance local contrast in the lung parenchyma, a common problem in overexposed radiographs. The feature matrix, 𝒯, is the output of Algorithm 1. Each row ${𝐫}_{i}$ corresponds to a single clinical radiograph and encapsulates the high-dimensional texture and statistical descriptors extracted from the lung parenchyma.

Traditional global intensity metrics often fail to distinguish between the focal opacities of bacterial pneumonia and the diffuse patterns of viral infections. While *EntropyMean* provides a global measure of textural disorder, the inclusion of *EntropyMax* and *Entropy90* allows the model to identify localised areas of high complexity typical of consolidations. *HazeRatio* and *Solidity* act as proxies for radiographic density. *Solidity* measures the ratio of high-intensity pixels (> 0.85) to capture the dense opacities of bacterial infiltrates, while *HazeRatio* identifies mid-intensity ground-glass textures common in viral cases.

The integration of the Minkowski-Bouligand *Fractal Dimension* (*D*) is also of importance [51]. Lung tissue naturally exhibits a branching, fractal-like structure. The progression of pneumonia disrupts this pattern by causing fluid accumulation and the accumulation of inflammatory cells. By utilising a log-log regression of box counts (*N*(*s*)) versus scale (*s*), we quantify the degree of structural fragmentation. In this way, a mathematically accurate descriptor of lung architecture is created, enabling the final diagnostic formula to distinguish between healthy, branching parenchyma and the disorganised, fragmented textures of infected tissue.

**Algorithm 1 MATLAB radiomic feature extraction pipeline**

**Require:** Set of raw CXR images ${ℐ}_{raw}$

**Ensure:** Structured radiomic feature matrix 𝒯

1: **for** each image $I\in {ℐ}_{raw}$ **do**

2:  **Geometry Check:** Calculate aspect ratio $AR=\frac{Width}{Height}$

3:  **if** *AR* < 0.7 **or** *AR* > 1.5 **then**

4:   Discard image (prevent geometric distortion)

5:  **end if**

6:  **Exposure Validation:** Calculate $\mu_{I}$ and $\sigma_{I}$

7:  **if** $\mu_{I}<10$ **or** $\sigma_{I}<5$ **then**

8:   Discard image (poor exposure/low contrast)

9:  **end if**

10: **Standardization:** Resize to 1024×1024 and convert to double precision [0, 1]

11: **ROI Masking:** Crop to central lung field (Rows: 15%−85%, Cols: 10%−90%)

12: **Enhancement:** Apply Contrast Limited Adaptive Histogram Equalisation (CLAHE)

13: **Texture Mapping:** Compute local entropy map $E_{map}$ using 9×9 neighborhood

14: **Density Extraction:**

• Compute *HazeRatio* via intensity thresholding [0.7τ,1.2τ]

• Compute *Solidity* (ratio of high-intensity pixels > 0.85)

15: **Spatial Statistics:** Extract Contrast and Homogeneity from Symmetric GLCM

16: **Structural Complexity:**

• Apply Canny edge detection

• Estimate Fractal Dimension *D* via Box-Counting method: $D=\lim_{s\to 0}\frac{\log N(s)}{\log(1/s)}$

17:  **Aggregation:** Append features to row vector ${𝐫}_{i}$

18: **end for**

19: Construct feature Table $𝒯=[{𝐫}_{1},{𝐫}_{2},\ldots ,{𝐫}_{n}{]}^{T}$ and export to CSV

### Genetic programming for symbolic classification

The objective of the SC framework is to discover a mathematical function *f*(*X*) that maps the radiomic feature space to a diagnostic label by evolving a population of candidate expressions. Unlike traditional classification (and regression), SC (and SR) does not assume a fixed model structure, but optimises both the functional form and the parameters simultaneously. Here, we have employed the PySR framework [52], which is based on a high-performance Julia-based backend to perform efficient symbolic search. The process utilises a tree-based representation of mathematical expressions, where internal nodes are operators (e.g., add, mul) and leaf nodes are radiomic features. The evolution process is described in Algorithm 2.

**Algorithm 2 Symbolic classification via Multi-population Regularised Evolution (PySR)**

**Require:** Radiomic Training Data $\left(X_{train},y_{train}\right)$, Number of populations *P*, Number of iterations *I*

**Ensure:** Best-fit diagnostic formula *f*(*X*) from the Pareto front

1: **Initialization:** Standardise $X_{train}$; initialize *P* independent populations of mathematical expressions using operators $\{+,-,\times ,÷,\log,\sqrt{\}}$.

2: **for** each iteration *i* = 1 **to** *I* **do**

3:  **Regularised Evolution:**

• Sample a small sub-population (tournament) from each island.

• Perform mutations and crossovers to generate new candidate programs.

• Replace the oldest individuals in the sub-population with new high-fitness offspring (Age-layered evolution).

4:  **Constant Optimisation:** Refine numerical constants in expressions using BFGS or similar gradient-based methods to minimize cross-entropy loss.

5:  **Migration:** Periodically exchange the highest-performing individuals between the *P* populations to maintain genetic diversity.

6:  **Simplification:** Apply automated algebraic simplification rules to reduce structural complexity.

7:  **Pareto Front Update:** Update the “Hall of Fame” with expressions that achieve the lowest loss for each discrete complexity level.

8: **end for**

9: **Model Selection:** Identify the optimal *f*(*X*) by selecting the expression on the Pareto front that maximises accuracy while satisfying the complexity constraint.

By utilising multi-population regularised evolution and Pareto-front optimisation, the framework identifies the most parsimonious mathematical expressions that maximise diagnostic accuracy. This search strategy effectively balances predictive power against structural complexity to prevent overfitting. It ensures that the final model is not only predictive but also clinically interpretable, providing a direct mathematical relationship between radiomic features and patient diagnosis.

## Results and discussion

### The derived symbolic expression

In this paper, the SC framework has been configured for a binary classification task, optimised to distinguish between “Normal” and “Pneumonia” (Bacterial and Viral pneumonia) cases. While the dataset distinguishes between bacterial and viral cases, the primary objective of the evolutionary process is to identify a single infection signal, regardless of the underlying pathogen, to simplify computation. Thus, the investigation becomes a binary classification problem. The symbolic evolution process yielded a parsimonious diagnostic expression that considers two main features and discards the rest (two of the ten available candidate descriptors). The formula for the diagnostic score *Z* is defined as follows:


$$\begin{array}{c} Z=\log(\alpha \cdot \text{FractalDim}-\beta \cdot \text{EntropyMean}+\gamma )+\delta \end{array}$$ (1)

where the constants range values are extracted from a 10-fold stratified cross-validation process, shown in Table 2. The best equation writes as: α=0.392, β=0.711, γ=1.966, and δ=0, leading to ACC = 0.908 and AUC = 0.959.

**Table 2:** Performance metrics and symbolic constants for the 10-fold cross-validation of the Symbolic Classifier. Each fold corresponds to the general form: log(α·FractalDim−β·EntropyMean+γ)+δ. Summary statistics (Mean ± SD) are provided in the final row.

Fold	ACC	AUC	α	β	γ	δ
Fold 1	0.891	0.944	0.390	0.710	1.964	0.000
Fold 2	0.873	0.941	0.527	1.000	2.747	−0.336
Fold 3	0.837	0.911	1.000	1.000	3.657	−0.628
Fold 4	0.875	0.931	0.385	0.720	1.966	0.000
Fold 5	0.893	0.951	0.392	0.715	1.963	0.000
Fold 6	0.861	0.912	0.450	1.000	2.141	0.000
Fold 7	0.872	0.932	0.392	0.715	1.965	0.000
Fold 8	0.908	0.959	0.392	0.711	1.966	0.000
Fold 9	0.864	0.947	0.390	0.718	1.892	0.000
Fold 10	0.828	0.888	1.000	1.000	3.415	−0.568
**Mean ± SD**	**0.870 ± 0.024**	**0.932 ± 0.021**	**0.532 ± 0.245**	**0.829 ± 0.141**	**2.371 ± 0.672**	**−0.153 ± 0.252**

*EntropyMean* captures local intensity disorder, which is reduced in regions of pneumonic consolidation because the affected lung becomes uniformly dense. The negative sign on *EntropyMean* is consistent with this. *FractalDim* captures texture self-similarity, which is disrupted by inflammatory infiltrate. The positive sign is consistent with reports linking fractal-dimension increase to parenchymal heterogeneity [53–55].

Equation 1 computes a diagnostic score *Z*, which integrates the radiomic inputs and represents the log-odds of infection. A higher positive *Z* value corresponds to a higher probability of pneumonia after the logistic transformation is applied, giving the final diagnostic probability *P*(Pneumonia):


$$\begin{array}{c} P=\frac{1}{1+e^{-Z}} \end{array}$$ (2)

By prioritising transparency/explainability over black-box DL frameworks, our SC approach ensures that the model’s logic remains accessible to the physician. In a high-stakes diagnostic setting, this interpretability is essential, as it empowers clinicians to manually verify the results, audit the logic for clinical consistency, and contest any automated findings that may contradict their professional expertise. Unlike black-box models that provide only a final prediction, our symbolic formula offers a transparent decision rule that may support clinician oversight and could facilitate integration into existing diagnostic workflows.

**Fig 2:**
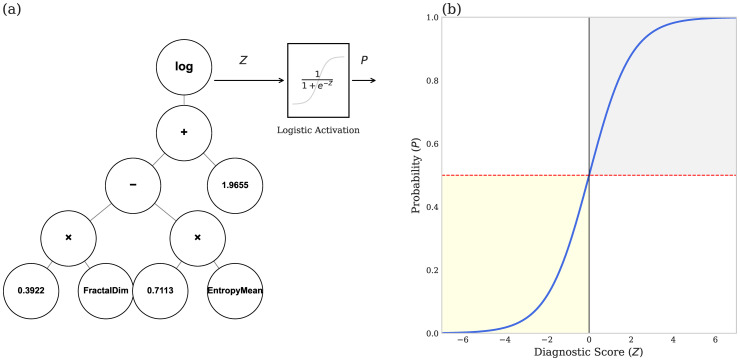
Symbolic classification. **(a)** Tree-based representation of the SC expression, **(b)** sigmoidal transformation of the diagnostic score, highlighting the *Z* = 0 decision boundary and the corresponding classification regions for “Normal” (green) and “Pneumonia” (pink) cases.

As shown in the computational tree of Fig 2(a), the two selected features are combined to generate a singular diagnostic score (*Z*). This value is then normalised via a logistic activation function (Fig 2(b)) to produce a continuous probability score *P*. The steepness of the sigmoidal curve around the *Z* = 0 intercept demonstrates the model’s ability to distinguish between healthy and pathological lung states. The yellow region (*Z* < 0) corresponds to “Normal” cases, while the grey region (*Z* > 0) corresponds to “Pneumonia”.

### Equation selection and accuracy

**Fig 3:**
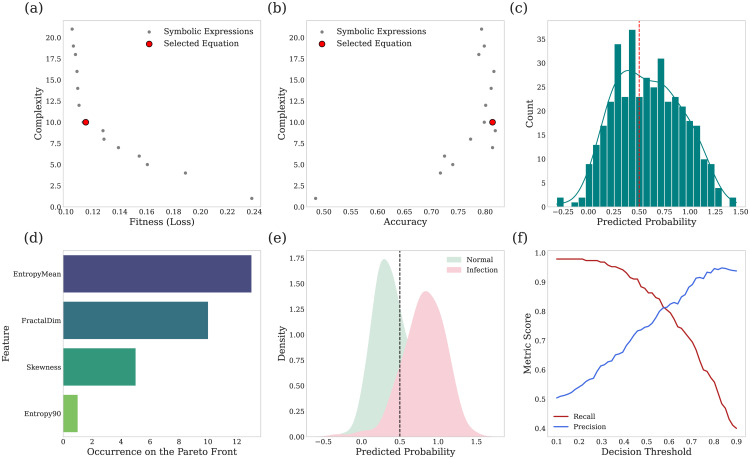
Diagnostic analysis of the proposed symbolic model on the Normal vs. Pneumonia binary task. **(a)** Pareto front of fitness (loss) vs. complexity. **(b)** Accuracy vs. complexity for the same Pareto-front members. The selected equation (red circle, complexity 10, loss ≈0.115) lies at the elbow of the front. **(c)** Distribution of predicted probabilities on the test set, centred near P≈0.6 with a broad single mode. **(d)** Frequency of feature occurrence across the Pareto front: *EntropyMean* and *FractalDim* dominate, while *Skewness* and *Entropy90* appear only sparsely in higher-complexity members. **(e)** Class-conditional probability density showing distinct separation between the “Normal” (green) and “Infection” (pink) populations, either side of the 0.5 decision threshold. **(f)** Precision–recall trade-off across the full range of decision thresholds; the curves intersect near 0.65.

The final diagnostic equation has been selected through a Pareto-front analysis that balances classification with mathematical complexity (Fig 3(a–b)). The symbolic search returns a family of expressions, each with increasing complexity, representing different operating points on the accuracy vs simplicity trade-off. The selection criterion is based on both predictive power and mathematical parsimony. Simple expressions are easier to use, interpret, and generalise [56], are less likely to overfit [57], and are typically more physically consistent than the over-parameterised representations that characterise DL architectures.

In Fig 3(a) (complexity vs. fitness), each grey dot is a unique symbolic expression returned on the Pareto front. Members on the left of the panel achieve very low loss but at very high complexity, while members on the right are simple but weaker in predictive performance. The selected equation (red dot) sits at the elbow of the curve. Thus, any further reduction in loss requires a disproportionate increase in complexity. Fig 3(b) presents the complexity vs. accuracy space. The chosen equation again occupies a region where additional complexity yields negligible accuracy gain. The equations that extrapolate to the upper end of the complexity spectrum of the front are harder to interpret and would increase the risk of overfitting. The selected equation from the 10-fold process (see Table 2) attains an average accuracy of [0.870±0.024] and an AUC of [0.932±0.021].

The selected expression is further characterised through its probability behaviour, feature usage, and threshold sensitivity (Figs 3(c–f)). The histogram in Fig 3(c) shows that predicted probabilities span roughly [0.4, 0.8] with a broad single mode around P≈0.6. Although individual probabilities are not maximally polarised, the class-conditional density in Fig 3(e) confirms that the model has learnt a genuine separation: “Normal” cases concentrate at lower probabilities (mode ≈0.57) while “Pneumonia” cases concentrate at higher probabilities (mode ≈0.72), with limited overlap around the 0.5 threshold. Together, Figs 3(c) and 3(e) are consistent with a calibrated decision boundary.

The feature-frequency plot in Fig 3(d) reveals that, across the entire Pareto front, only four of the ten radiomic features are considered. *EntropyMean* and *FractalDim* dominate the high-performance region of the front, *Skewness* appears only in a few intermediate-complexity members, while *Entropy90* appears sparsely. The remaining six features (*EntropyMax*, *Haze*, *Solidity*, *Contrast*, *Homogeneity* and *Kurtosis*) are not selected by the search at any complexity. The convergence is observed only on *EntropyMean* (a global measure of textural disorder) and *FractalDim* (a measure of texture self-similarity). Pneumonic consolidation produces uniformly dense regions with lower local entropy, while an inflammatory infiltrate disrupts the lung’s regular branching pattern and increases its fractal dimension. The fact that the parsimony-driven symbolic search rediscovers this two-feature substructure without any external feature-importance step indicates that the relationship is an inherent property of the data.

Finally, the precision–recall trade-off in Fig 3(f) traces the full operating curve of the selected equation. Recall remains close to 1 for thresholds up to ≈0.55 before falling rapidly, while precision rises from its base rate to nearly 1 between thresholds 0.5 and 0.7. The two curves cross at approximately 0.65, indicating an operating point where sensitivity and positive predictive value are both close to 0.85. This balance is desirable in a clinical context where both false negatives (missed pneumonia) and false positives (unnecessary imaging or follow-up) are not desired.

### Comparative analysis with black-box classifiers

After employing SC methods for binary classification to distinguish between “Normal” and “Pneumonia” cases, an additional step is required to accurately differentiate among the three distinct classes in the Kermany Pediatric CXR dataset, i.e., “Normal”, “Bacterial”, and “Viral”. The extracted radiomic features have been evaluated using four standard ML architectures, i.e., Support Vector Machines (SVMs) with a Radial Basis Function (RBF) kernel, Logistic Regression (LR), Random Forest (RF), and XGBoost (XGB). These models are characterised as black-box due to their high dimensionality and the non-intuitive nature of their internal decision boundaries.

The algorithms have all been tuned to run with optimal hyper-parameters, and the comparison results, including the mean score, standard deviation, and approximate 95% confidence intervals (CI), are summarised in Table 3. On the binary task (Normal vs. Pneumonia), the four black-box classifiers lie within a narrow performance band. XGBoost has the highest mean accuracy of 0.92±0.02 (95% CI: [0.89, 0.95]) and an AUC of 0.97±0.01, while RF, SVM (RBF) and LR fall within one percentage point and within overlapping confidence intervals. The SC, despite being constrained to a single closed-form expression, achieves 0.87±0.02 accuracy (95% CI: [0.84, 0.91]) and an AUC of 0.93±0.02 (95% CI: [0.90, 0.96]), remaining within five percentage points of the strongest ensemble while offering full mathematical transparency. The accuracy scores for all black-box classifiers decrease on the ternary task (Normal / Bacterial / Viral). Random Forest leads at 0.78±0.02 (95% CI: [0.74, 0.81]), followed by XGBoost and SVM (RBF) at 0.76, and LR at 0.73. This drop confirms that the textural overlap between bacterial and viral pneumonia is harder to resolve than the normal/infected decision. Conclusively, we observe that the radiomic feature set generalises well across model families for the binary task, whereas errors arise when the models are asked to distinguish between the two pneumonia subtypes. The proposed SC achieves a competitive accuracy/AUC trade-off on the binary task while preserving the full interpretability that motivates its use.

**Table 3:** Combined binary (Normal vs. Pneumonia) and ternary (Normal / Bacterial / Viral) classification performance of various ML classifiers and the Symbolic Classifier (SC), with variance estimates and 95% confidence intervals on 10-fold cross-validation.

Model	Binary Acc.	Binary AUC	Ternary Acc.
**XGBoost**	0.918±0.015	0.969±0.009	0.762±0.015
	[0.889, 0.947]	[0.951, 0.987]	[0.733, 0.791]
**Random Forest**	0.914±0.014	0.967±0.008	0.779±0.018
	[0.887, 0.941]	[0.951, 0.983]	[0.744, 0.814]
**SVM (RBF)**	0.914±0.013	0.965±0.010	0.756±0.013
	[0.888, 0.939]	[0.945, 0.985]	[0.731, 0.781]
**Logistic Regression**	0.903±0.009	0.958±0.009	0.727±0.011
	[0.885, 0.921]	[0.940, 0.976]	[0.705, 0.749]
**Symbolic Classifier**	0.870±0.024	0.932±0.021	—
	[0.835, 0.905]	[0.901, 0.963]	

**Fig 4:**
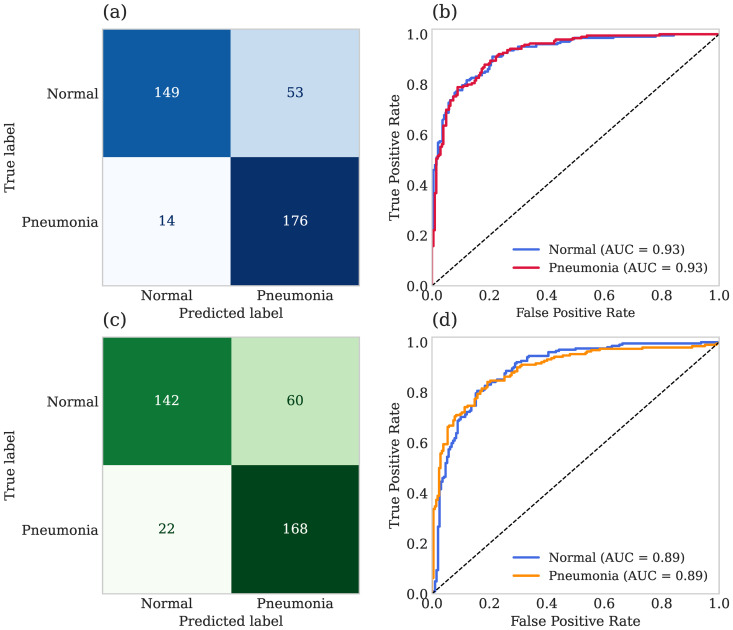
Confusion matrices and ROC plots for the two best-performing models. **(a)-(b)** XGBoost and **(c)-(d)** SC.

Next, we retain the best-performing algorithm for the binary task, XGBoost, and compare it with the symbolic expression proposed in Eq. 1 in Fig 4. The classification performance is evaluated with confusion matrices (CMs) and Receiver Operating Characteristic (ROC) curves.

Fig 4(a) presents the CM for the binary XGBoost model (“Normal”, “Pneumonia”). The diagonal elements represent the true positives for each class: 149 for “Normal”, and 176 for “Pneumonia”. The off-diagonal elements indicate relatively low misclassification rates. This high degree of class separation is reflected in Fig 4(b), where the ROC curves for both classes show excellent ability to differentiate between the two cases. The Area Under the Curve (AUC) value is 0.93, indicating that the XGBoost ranks positive instances higher than negative ones across a wide range of decision thresholds.

Fig 4(c) displays the CM for the binary SC model. The model correctly identifies 142 “Normal” and 168 “Pneumonia” cases, resulting in an overall accuracy of


$$\begin{array}{c} \text{Accuracy}=\frac{142+168}{142+60+22+168}\approx 79.1\%. \end{array}$$

We have seen that the cross-validated mean accuracy is [0.870±0.024] (10-fold, training partition). When the SC formula is applied to the independent hold-out test set, the accuracy decreases to 79.1%, consistent with the loss of fold-specific re-fitting. The achieved value is fine for a simplified symbolic model. The corresponding ROC curve in Fig 4(d) gives an AUC of 0.89, which is slightly smaller than the one achieved by the XGBoost (0.93). This is due to the overlap in the *z*-score distributions. While the specific threshold *z* > 0 yields a high accuracy point on the curve, the AUC measures the model’s performance across all possible thresholds.

However, the clinical utility of this symbolic model should be evaluated based on its transparency rather than solely through performance metrics such as AUC. While common DL models frequently report accuracies of 99% on the Kermany Pediatric CXR dataset [3,4,6,7,39], these architectures serve as black boxes, offering no insight into the radiological features that drive diagnoses. In high-stakes medical environments, a marginal increase in accuracy does not necessarily outweigh the risks of opaque decision-making, in which models may inadvertently rely on spurious correlations or image artefacts. In contrast, our symbolic approach provides an explicit, human-readable mathematical formula that specifies how morphological descriptors interact to produce a diagnostic score. This deterministic clarity allows clinicians to verify that the model is grounded in actual pulmonary pathophysiology. Therefore, while the XGBoost or DL ensembles may offer superior statistical separation, the symbolic model provides a level of scientific accountability and interpretability that is a prerequisite for the safe and ethical deployment of AI in clinical practice.

### Comparative analysis with state-of-the-art methods

Table 4 compares the proposed SC with the principal pneumonia-detection methods reported on the Kermany Pediatric CXR benchmark [39], per architectural family. The first single-model CNN baselines are mainly paired with existing ImageNet [67] pretrained backbones. Starting from the original InceptionV3 [68] pipeline [39], followed by a lightweight custom CNN [58], a residual-network transfer-learning model [59], and the DenseNet [69] transfer-learning pipeline [6]. Each progressively deeper architecture yields a small accuracy gain, but interpretability is provided uniformly via post-hoc saliency methods (typically Grad-CAM), and the underlying decision rule remains an opaque parametric mapping of 10^6^–10^7^ weights. Ensemble approaches have shown even higher accuracy. A five-model ensemble combines predictions from AlexNet [70], DenseNet121 [69], InceptionV3 [68], ResNet18 [71], and GoogLeNet [72] to reach 96.4 % accuracy [60], while another weighted ensemble reports 98.4 % [61]. They both exceed $5\;\times \;10^{7}$ aggregate trainable parameters and again rely on Grad-CAM for explanation, reproducing the same accuracy/transparency profile as the single-network baselines but at greater inference cost.

**Table 4:** Comparison of representative pneumonia-detection methods evaluated on the same pediatric chest X-ray benchmark [39] with the proposed SC. Parameter counts are approximate and reflect the standard reference implementations of each architecture. Intrinsic interpretability denotes a model whose decision rule is itself the explanation, whereas Post-hoc indicates that interpretability is supplied through a separate approximation tool.

Reference (year)	Method	Params.	Interpretability	Closed-form	Acc. (%)
[39] (2018)	InceptionV3 transfer learning	∼24 M	Post-hoc (saliency)	No	92.8
[58] (2019)	Custom CNN, four convolutional blocks	∼5 M	None reported	No	93.7
[59] (2020)	ResNet + transfer learning	∼25 M	Post-hoc (Grad-CAM)	No	96.7
[6] (2020)	DenseNet201 transfer learning	∼20 M	Post-hoc (Grad-CAM)	No	98.0
[60] (2020)	Ensemble of five pretrained CNNs	>50 M	Post-hoc (Grad-CAM)	No	96.4
[61] (2020)	Weighted DL ensemble	>50 M	Post-hoc (Grad-CAM)	No	98.4
[62] (2020)	Capsule network (CapsNet)	∼11 M	Post-hoc (CapsNet activ.)	No	95.9
[63] (2020)	Multi-dilation CNN (CovXNet)	∼11 M	Post-hoc (Grad-CAM)	No	98.1
[64] (2022)	Hybrid CNN + Vision Transformer (IEViT)	∼87 M	Post-hoc (attention maps)	No	96.4
[65] (2022)	Multi-task Vision Transformer	∼86 M	Post-hoc (attention maps)	No	95.5
[66] (2023)	MedViT (medical Vision Transformer)	∼30 M	Post-hoc (attention rollout)	No	96.7
XGBoost	Radiomic features + XGBoost	∼10^2^	Feature-level	No	92.0
**SC**	**Radiomic features + GP**	$\sim 10^{1}$	**Intrinsic**	**Yes**	**79.0-87.0**

Architectures outside the standard CNN approaches have also been evaluated on the same benchmark, such as a capsule network with dynamic routing [62] and CovXNet [63], a multi-dilation CNN with transferable multi-receptive-field features, both with Grad-CAM explainability. Some recent methods employ self-attention, including the hybrid CNN/Vision Transformer [64], the multi-task Vision Transformer [65], and the medical Vision Transformer [66]. While these architectures advance both accuracy and adversarial stability, the form of interpretability they offer (i.e., attention-based saliency) remains a post-hoc approximation of the underlying 10^7^–10^8^-parameter decision function.

The reported binary accuracy of the proposed SC model ranges from 79% on the independent test set to is lower than that of competing DL pipelines, the proposed model differs from every entry in Table 4 by several orders of magnitude in parameter count, replaces a separate post-hoc explanation step with an intrinsically interpretable closed-form expression, and is, therefore, the only one whose decision rule is fully auditable without recourse to an approximation tool. We emphasise that the SC framework is presented not as a replacement for these high-performing architectures, but as a complementary, intrinsically interpretable alternative that broadens the methodological repertoire available for clinically transparent diagnosis.

### External validation on independent chest X-ray datasets

Next, the extrapolation ability of the chosen SC expression is investigated. The external set is taken from the NIH ChestX-ray14 database [73], which was first introduced in [8], and is publicly released with no usage restrictions for research and educational purposes. This release includes frontal-view, 1024×1024, 8-bit grey-scale PNG chest radiographs of 30,805 unique adult patients collected at the U.S. National Institutes of Health Clinical Centre between 1992 and 2015. Apart from normal, the dataset employs disease image labels such as *Atelectasis, Cardiomegaly, Consolidation, Edema, Effusion, Emphysema, Fibrosis, Hernia, Infiltration, Mass, Nodule, Pleural Thickening, Pneumonia, Pneumothorax*. To test our approach, we selected a stratified random sample of *n* = 60 images with the *Pneumonia* label (positive class), and *Normal* controls from the same release. Radiomic features were re-extracted from the original 1024×1024 PNGs through the Algorithm 1 pipeline, without resizing.

NIH ChestX-ray14 differs from our original training set (Kermany Pediatric CXR) as it refers to an adult population whose thoracic anatomy, lung-volume distribution, and the pathology profile differ from the pediatric pool used to derive Eq. 1 [74]. We, therefore, expect that the absolute magnitudes of (α,β,γ,δ) in Eq. 1 need re-calibration. To this end, we keep the structural form of Eq. 1 fixed and re-estimate its four numeric constants (α,β,γ,δ) on the NIH ChestX-ray14 images via differential evolution against a binary cross-entropy objective with the same decision rule used in cross-validation (Z≥0.5).

Comparison results are tabulated in Table 5.

**Table 5:** External validation of Eq. 1 with the NIH ChestX-ray14 dataset. Constants were refit by differential evolution on the external set. The symbolic form is held fixed.

Dataset	α	β	γ	δ	Acc	AUC	F1
Kermany Pediatric CXR	0.3922	0.7113	1.9655	0.0000	0.870	0.932	0.870
NIH ChestX-ray14	17.8540	6.1727	0.7722	−0.9049	0.717	0.661	0.191

On the NIH ChestX-ray14 subset, the re-calibrated equation reached an accuracy of 0.717 and an AUC of 0.661. The remaining metrics reveal an asymmetric error pattern. For the fixed Z≥0.5 decision threshold, the rule is conservative on adult data, since the few cases it commits to are mostly correct, but most positive cases are missed. The AUC of 0.661, by contrast, summarises the equation’s ability to rank pneumonia cases higher than healthy ones, and shows that the symbolic form retains its discriminative information on adult radiographs. Moreover, the re-calibrated constants reverse the relative weight of the two features compared with the pediatric fit. The optimiser assigns the dominant contribution to *FractalDim* and a much smaller relative weight to *EntropyMean* for the adult radiographs. This is consistent with the larger, denser bony anatomy in adult chest radiographs, which amplifies self-similar structural content. The difficulty to impose the same method for these two datasets has been also investigated in [75], where a CNN network achieved 0.985 ACC and 0.998 AUC for the Kermany Pediatric CXR and 0.721 ACC and 0.787 AUC for the NIH ChestX-ray14 dataset.

Conclusively, the relationship between textural disorder and self-similarity uncovered by SC is not an artifact. The optimal constants for the general form of Eq. 1 seem to be dataset-specific. Thus, the symbolic expression poses as a re-calibratable rule and cannot be seen as a general plug-and-play rule. As the absolute performance on adult data is lower than on the pediatric data, the results offer preliminary indication that the symbolic form may be portable, with the understanding that any practical use on a new dataset would require dataset-specific re-calibration of (α,β,γ,δ) and, ideally, expert-reviewed labels.

## Conclusion

This paper demonstrates that pneumonia detection in chest radiographs can be achieved effectively using a transparent, feature-based pipeline. By integrating targeted image processing with symbolic classification, we discovered a non-linear mathematical expression that achieves up to 87.00% accuracy, providing a verifiable white-box alternative to traditional diagnostic models. The expression reflects the physical fact that pneumonia replaces the lung’s complex, branching air sacs with solid, uniform infiltrates. By mathematically contrasting the increase in opacity against the decrease in structural fragmentation, it identifies the infectious signal within the high-dimensional radiomic space. The proposed intrinsic interpretable framework has been compared with a multi-class XGBoost model and existing deep learning models from the literature (mainly black-box or post-hoc explainable).

These findings highlight that texture-based image features, such as *FractalDim* and *EntropyMean*, are the most critical descriptors for identifying pulmonary infection and form the core of an interpretable diagnostic pipeline. By evolving a model from physically grounded texture descriptors rather than raw pixel intensities, and subjecting the results to robustness checks, we aim for diagnostic outputs that reflect reasonably stable image-based features associated with pneumonia and that may be less susceptible to the failure modes commonly observed when black-box models encounter out-of-distribution data, such as hospital-specific artifacts or differences in patient positioning.

Apart from interpretability, symbolic models offer distinct advantages in clinical radiology through their inherent auditability and parsimonious deployment. Notwithstanding their high performance, modern deep learning architectures can be extremely resource-intensive, and their re-calibration to a new clinical site typically requires retraining or domain adaptation at non-trivial computational cost. In contrast, the proposed model is expressed as a simple closed-form mathematical expression with only four numeric constants and offers significant flexibility through its tunable decision threshold. The additional external validation experiment on the NIH ChestX-ray14 adult dataset further showed that the symbolic form retains discriminative ability, after proper re-calibration. Together, these properties make the proposed classifier computationally lightweight, easily integrable into edge devices or low-resource clinical environments without requiring high-end hardware, and straightforward to re-calibrate to local patient cohorts when site-specific data become available.

However, several limitations must be acknowledged before considering clinical translation. The proposed model should be interpreted as a research-level, intrinsically interpretable baseline. Its clinical usefulness remains untested and requires prospective pediatric validation, radiologist comparison, calibration analysis, and workflow evaluation.
